# Bilateral Coats' Disease Combined with Retinopathy of Prematurity

**DOI:** 10.1155/2015/364395

**Published:** 2015-08-30

**Authors:** Huseyin Gursoy, Nazmiye Erol, Mustafa Deger Bilgec, Hikmet Basmak, Ozden Kutlay, Huseyin Aslan

**Affiliations:** ^1^Department of Ophthalmology, Eskişehir Osmangazi University Medical Faculty, 26180 Eskişehir, Turkey; ^2^Department of Medical Genetics, Eskişehir Osmangazi University Medical Faculty, 26180 Eskişehir, Turkey

## Abstract

*Purpose*. To report a case of bilateral Coats' disease combined with retinopathy of prematurity (ROP). *Case*. Retinal vascularization was complete in the right eye, whereas zone III, stage 3 ROP and preplus disease were observed in the left eye at 43 weeks of postmenstrual age (PMA) in a 31-week premature, 1200-g neonate. Intraretinal exudates developed and retinal hemorrhages increased in the left eye at 51 weeks of PMA. Diode laser photocoagulation (LP) was applied to the left eye. Exudates involved the macula, and telangiectatic changes developed one month following LP. Additional LP was applied to the left eye combined with intravitreal bevacizumab (IVB) injection at 55 weeks of PMA. Disease regressed one month after the additional therapy. At the 14-month examination of the baby, telangiectatic changes and intraretinal exudates were observed in the right eye. Diode LP was applied to the right eye combined with IVB injection. Exudates did not resolve completely, and cryotherapy was applied one month following LP. Retinal findings regressed three months following the cryotherapy. *Conclusion*. This is the first report of presumed bilateral Coats' disease combined with ROP. If Coats' disease could be diagnosed at early stages, it would be a disease associated with better prognosis.

## 1. Introduction

Coats' disease is a retinal disorder associated with telangiectasias which consist of dilated capillaries and microaneurysms and exudation. The majority of cases are diagnosed in males around the age of ten. The retinal findings are usually unilateral, although some bilateral cases with prematurity have been presented [1]. Three infants, who were diagnosed with unilateral Coats' disease, have been reported [2–4]. The aim our paper is to report a case of presumed bilateral Coats' disease combined with retinopathy of prematurity (ROP) and treated with intravitreal bevacizumab injection (IVB), laser photocoagulation (LP), and cryotherapy. To the best of our knowledge, this is the first report of presumed bilateral Coats' disease combined with ROP.

## 2. Case Report

Written informed consent was procured from the parents of the patient prior to therapy and also for the publication of this case report. All interventions, namely, 810-nm diode LP (Iridex, Mountain View, CA), IVB (Avastin; Genentech, San Francisco, CA) injection, and cryotherapy, were performed under general anesthesia. Fundus photographs were taken using the RetCam (Clarity Medical Systems, Pleasanton, CA).

**Figure 1:**
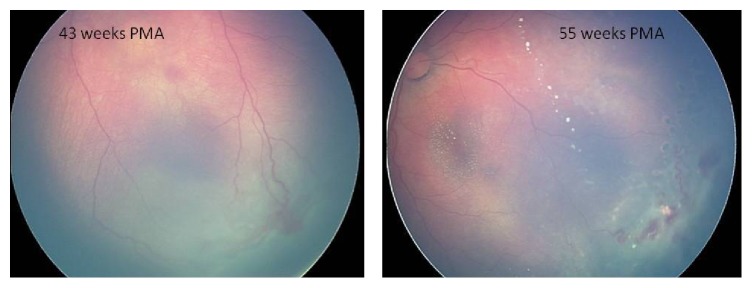
RetCam photograph of the left eye, showing zone III, stage 3 ROP and exudates involving the macula and a telangiectatic vascular appearance in the temporal quadrant at 43 and 55 postmenstrual ages, respectively.

The 31-week premature, 1200-g neonate was hospitalized in our institution's neonatal intensive care unit (NICU) and treated with nasal continuous positive airway pressure as primary respiratory supporting system for three weeks. The infant had no family history nor did the case have any associated systemic findings. The initial examination for ROP was performed at 6 weeks of age by indirect ophthalmoscopy after the infant was discharged from the NICU. Zone III, stage 1 ROP was observed in the right eye, and zone II, stage 1 ROP was seen in the left eye. Weekly examinations were performed to follow up the retinal vascularization. Retinal vascularization was almost complete in the right eye, whereas zone III, stage 3 ROP and preplus disease were observed in the left eye at 43 weeks of postmenstrual age (PMA) (Figure 1). Intraretinal exudates developed and retinal hemorrhages increased in the temporal quadrant of the left eye at 51 weeks of PMA. Diode LP was applied to the left eye on the day after these findings were observed. The left eye received a near confluent pattern of diode LP (duration = 300 ms; laser power = 400 to 700 mW; 1,550 shots) to the avascular retina. During follow-ups the preplus appearance persisted, and exudates involved the macula and a telangiectatic vascular appearance including microaneurysms developed in the temporal quadrant one month following LP (Figure 1). Additional diode LP was applied around the telangiectatic vessels in the left eye combined with IVB injection at 55 weeks of PMA. Bevacizumab 0.75 mg (0.03 cc) was injected intravitreally using a 30-gauge needle placed 1 mm behind the limbus. The amount of bevacizumab was preferred according to our results obtained from our previous applications in ROP cases [5]. Weekly examinations were performed throughout the first month after IVB injection. Exudates and the vascular activity resolved one month after the additional therapy.

**Figure 2:**
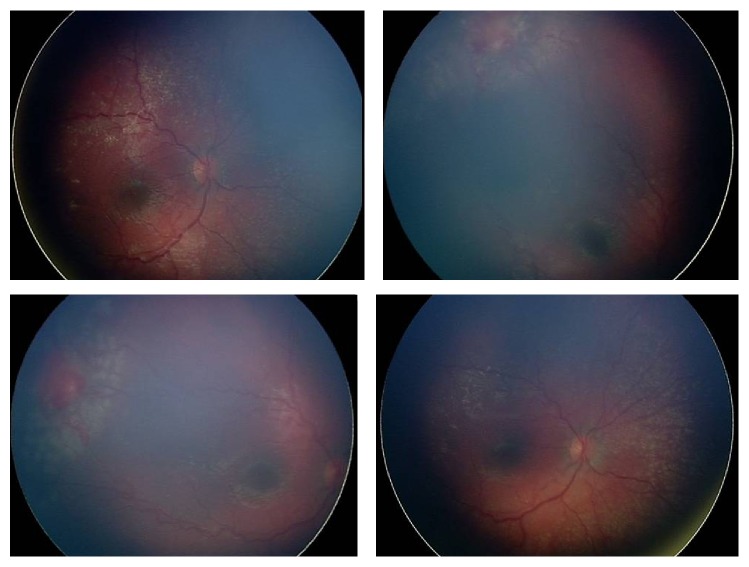
RetCam photograph of the right eye just after laser treatment at the 14-month examination of the baby, showing telangiectatic vascular appearance in the temporal quadrant and exudates.

**Figure 3:**
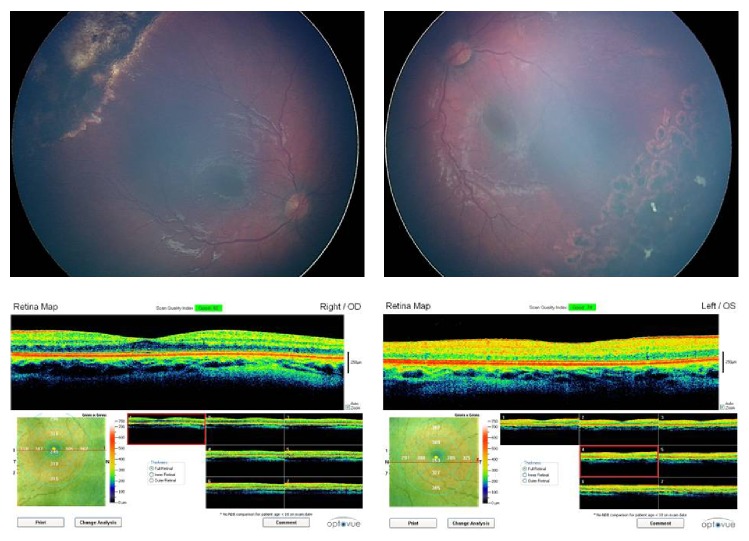
The regressed disease in both eyes and the OCT maps at three years of age.

Monthly examinations were performed. Total regression of the telangiectatic changes and the exudation in the left eye was documented. At the 14-month examination of the baby, telangiectatic changes including microaneurysms were observed in the temporal quadrant of the right eye. Intraretinal exudates developed in the temporal and nasal quadrants of the right eye. Diode LP (300 ms; laser power = 400 to 700 mW; 350 shots) was applied around the area of telangiectatic retinal changes in the right eye combined with IVB injection two days after the observation of the findings (Figure 2). Weekly examinations were performed throughout the first month after the treatment of the right eye. Exudates did not resolve completely and cryotherapy was applied to the area of telangiectatic retinal changes one month following LP. Total regression of the retinal findings occurred three months following the cryotherapy. At the final visit at the age of three years, spectral-domain optical coherence tomography (SD-OCT) (iVue, Optovue, Fremont, USA) images, cycloplegic refraction (handheld autorefractometer Retinomax (Nikon corporation, Tokyo, Japan)), and fundus photographs were obtained under general anesthesia (Figure 3). Fundus photographs showed the regression of the disease in both eyes. The SD-OCT map showed a well-formed foveal pit in both eyes. The eyes were orthotropic. The final cycloplegic refraction in spherical equivalent was +3.00 in the right and +2.00 in the left eye. The best corrected visual acuity was at least 0.5 in the right and 0.4 in the left eye.

### 2.1. Genetic Analysis

Genomic DNA was extracted from the peripheral blood sample by using MagNA Pure Compact DNA isolation kit and MagNA Pure LC instrument (Roche Applied Science) according to the manufacturers' instructions. Forward and reverse primers were obtained from the studies by Black et al. [6] and Kondo et al. [7]. PCR reactions were carried out in a final volume of 50 *μ*L (1X PCR buffer; 2 mM Mg^++^, 150 *μ*lM dNTPs, 5 pmol of each primer, 1.5 U Vivantis Taq, and 100 ng of genomic DNA). The following cycle was applied: 2 min at 95°C, 35 cycles of 20 s denaturation at 94°C, 30 seconds at 54°C (F1-R1) to 56°C (F2-R2), 57.5°C (F3-R3), 1 min extension at 72°C, and 7 min final extension at 72°C. Sequencing reactions for all exons of NDP gene were performed after ExoSAP purification (Affymetrix, HT ExoSAP-IT, High-Throughput PCR Product Cleanup). Sequence variations were analyzed at National Center for Biotechnology Information. The DNAs from the patient were analyzed for mutations in three exons and flanking exon–intron boundaries of the NDP gene. In the coding region and noncoding region there was no mutation.

## 3. Discussion

Most children with Coats' disease do not have any complaints, so the cases are usually diagnosed at late stages [8]. Early stages of Coats' disease are characterized by telangiectasias and exudation without retinal detachment [1]. We recognized the present case incidentally before the development of retinal detachment. Fundus examination is usually sufficient to diagnose Coats' disease [1]. We considered the case as Coats' disease after the recognition of exudation and vascular changes in the left eye, because these findings are not associated with ROP. The observation of similar findings in the right eye at the 14-month examination made us think of bilateral Coats' disease. 95% of Coats' disease is unilateral [9], but the other eye was also involved in the 14-month examination of our case.

Somatic mutations in the NDP gene have been implicated in Coats' disease [6, 7]. These mutations have been demonstrated in retinas of enucleated eyes from Coats' cases [6]. It was not possible to extract DNAs from the retina of this case, so blood samples of the family were used for genetic analysis. We thought that somatic mutations that occurred in early embryological life could have been found in peripheral blood of this patient, because the presentation was during first years of life. There was no mutation, but this does not exclude the diagnosis of Coats' disease.

The differential diagnosis of peripheral exudative retinal vascular lesions includes ROP, Coats' disease, familial exudative vitreoretinopathy (FEVR), and Norrie's disease (ND) [6, 7, 10]. Lipid exudates may develop in FEVR, but the cases are usually healthy term infants with a positive family history, associated with bilateral peripheral retinal avascularity seen temporally [10]. FEVR cannot be excluded based on these findings, because X-linked recessive inheritance and FEVR cases in premature infants have been reported [11, 12]. ROP also developed in our case with typical ridge formation, but the other findings in both eyes were typical for Coats' disease. ND is often associated with hearing loss and/or developmental delays. Symmetrical involvement and poor outcomes are other features of ND [13]. The lack of family history, the presence of telangiectatic changes, and prematurity supported the diagnosis of bilateral Coats' disease combined with mild ROP.

IVB injection, LP, and cryotherapy are different treatment choices in Coats' disease [14]. We preferred to use IVB injection and LP in both eyes, whereas cryotherapy was applied to the right eye. The incidental finding of Coats' disease saved the child's vision, but lifetime follow-up is required, since the disease can recur in years [15].

Based on our experience of this case, the subclinical retinal pathology in Coats' disease may develop at a very early age, but late diagnosis may be the major problem in many cases [16]. If Coats' disease could be diagnosed at early stages, it would be a disease associated with better prognosis.
